# Chikungunya fever and lymphedema of limbs

**DOI:** 10.1590/1677-5449.190100

**Published:** 2020-08-07

**Authors:** Beuy Joob, Viroj Wiwanitkit, Marcos Arêas Marques

**Affiliations:** 1 Private Academic Consultant, Bangkok, Thailand.; 2 Dr. D. Y. Patil Vidyapeeth University, Pune, India.; 1 Universidade do Estado do Rio de Janeiro – UERJ, Unidade Docente Assistencial de Angiologia, Rio de Janeiro, RJ, Brasil.

Dear Editor,

We read the publication on “Secondary lymphedema of limbs and chikungunya fever (CF)” with great interest.^1^ Marques et al. described “*the case of a patient who developed lymphedema of upper and lower limbs after an episode of CF*.^1^” Marques et al. also noted that “*the CF outbreak that occurred during 2015 and 2016 resulted in the first cases described in the medical literature of acute and chronic vascular complications secondary to infection by this arbovirus*.^1^” In fact, the vascular defect due to CF is rarely mentioned in the literature. Nevertheless, this is not the first report regarding CF and lymphedema. Lymphedema of the limbs in CF is also described in many previous reports from India.^2^^-^4

## RESPONSE LETTER

Dear Editor,

Thank you very much for your comment and for the timely and pertinent correction of the facts. Undoubtedly lymphedema had already been described by several Indian and Pakistani authors^1^^-^5, some cited in the letter to the editor, because it is an endemic infection in this region of the planet. However, in my article I draw attention to the Chikungunya epidemic in the South American continent, and particularly in Brazil, in the years 2015 and 2016, which drew the attention of many doctors, because until then it was an infection unknown to most professionals of health in Brazil.

In addition, after a new and extensive literature review and search in PubMed associating the term Chikungunya with the terms lymphedema, lymphoscintigraphy, vascular complications, or nuclear medicine^6^^-^12, I found no article associating lymphedema or lymphadenopathy secondary to Chikungunya virus infection and abnormal images of lymphoscintigraphy, as described in my article, including the references cited in the letter to the editor. Finally, and to be fair, the first reports, similar to the case report cited, described in Brazil, were made by researchers from the Federal University of Pernambuco in 2016, which even served as an incentive for my deepening of the subject.

## References

[1] Marques MA, Milhomens ALM, Vieira JM, Cardoso FR, Guedes HJ (2019). Secondary lymphedema of limbs and chikungunya fever. J Vasc Bras.

[2] Inamadar AC, Palit A, Sampagavi VV, Raghunath S, Deshmukh NS (2008). Cutaneous manifestations of chikungunya fever: observations made
during a recent outbreak in south India. Int J Dermatol.

[3] Bandyopadhyay D, Ghosh SK (2010). Mucocutaneous manifestations of Chikungunya fever. Indian J Dermatol.

[4] Rampal, Sharda M, Meena H (2007). Neurological complications in Chikungunya fever. J Assoc Physicians India.

